# Editorial: Neural pathophysiological mechanisms of neuropsychiatric disorders underlying substance abuse

**DOI:** 10.3389/fncel.2023.1194734

**Published:** 2023-04-25

**Authors:** Zhuo Wang, Cihang Gu, Pingming Qiu

**Affiliations:** ^1^School of Medicine, South China University of Technology, Guangzhou, China; ^2^School of Forensic Medicine, Southern Medical University, Guangzhou, China

**Keywords:** substance use disorder, neurological and psychiatric symptoms, addiction, neuron, brain

Substance abuse is often associated with neuropsychiatric disorders, which can complicate treatment and increase the risk of adverse outcomes. Recent advances in the understanding of the neural pathophysiological mechanisms underlying substance abuse and neuropsychiatric disorders have provided new insights into the development and maintenance of these conditions. At the cellular and molecular level, substance abuse, and neuropsychiatric disorders involve complex interactions between various brain regions and neurotransmitter systems. Dysregulation of these systems can lead to alterations in neural plasticity, gene expression, and synaptic transmission, ultimately leading to alterations in behavior and neural function. This complexity has posed significant challenges to the development of effective treatment strategies. Despite these challenges, researchers are making progress in understanding the neural pathophysiological mechanisms underlying substance abuse and neuropsychiatric disorders. By elucidating the mechanisms that drive these conditions, researchers are identifying new targets for drug development and novel treatment approaches.

The Research Topic “*Neural Pathophysiological Mechanisms of Neuropsychiatric Disorders Underlying Substance Abuse*” consists of six original research papers. In this Research Topic, we aim to consolidate the advances in the field of neural pathophysiological mechanisms underlying substance abuse and neuropsychiatric disorders.

Liang et al. investigate whether mid-to-late adolescent exposure to methamphetamine (METH) leads to long-lasting memory impairment. This study indicates that mice exposed to METH in mid-to-late adolescence have impaired memory ability in their adulthood. This may be the result of abnormal changes in the structural plasticity of the dorsal hippocampus. The causal relationship between changes in synaptic structural plasticity and memory impairment needs to be further confirmed.

Structural plasticity changes in the brain are thought to underlie, at least partially, drug-induced persistent changes in behavior. Drug administration induces persistent changes in the structure of the brain (i.e., length and branches of dendrites, the density of spine cells, and so on; Zhang et al., 2006; Bonilla-Del Rio et al., 2021), which was thought to restructure associated neuronal connection and contribute to drug addiction. Wang et al. suggest that METH induced distinct changes of dopaminergic and glutamatergic synapses and cells in the nucleus accumbens shell (NAcsh) of mice, which was blocked by the knockout of the dopamine D3 receptor gene, and may contribute to, at least partially, METH-induced behavior sensitization as well as the modulating effect of the dopamine D3 receptor.

NAc is also involved in the expression of cocaine addictive phenotypes, including acquisition, extinction, and reinstatement. Chen et al. look into the role of GRIP1 (Glutamate receptor-interacting protein 1) in D1-medium spiny neurons (MSNs) and D2-MSNs of the NAc in cocaine acquisition and reinstatement. This study identifies that GRIP1 downregulation in D1-MSNs has a positive effect on cocaine acquisition and reinstatement, while GRIP1 downregulation in D2-MSNs has a negative effect. Additionally, GRIP1 downregulation in D1-MSNs plays a leading role in cocaine acquisition and reinstatement.

Zhang et al. discuss the associations of heroin-memory reconsolidation with cAMP-dependent protein kinase A (PKA) of BLA based on the fundamental effect of PKA in synaptic plasticity and memory process. The researchers conclude that PKA inhibition disrupts the reconsolidation of heroin-associated memory, reduces subsequent drug seeking, and prevents relapse, which is retrieval-dependent, time-limited, and BLA-dependent. Their results indicate that PKA inhibition may have potential therapeutic effects in heroin abuse disorders.

In the central nervous system, METH functions as an enhancer for monoamine neurotransmitter release (Wang et al.). METH has been also proven to regulate the synaptic structure and different METH dosages could differently regulate synaptic plasticity (Yan et al., 2019). Ding et al. study the effects of a low dose and a high dose of METH on synaptic structure alternation in the hippocampus and prefrontal cortex (PFC) to reveal the underlying mechanism involved in the process. Their results indicate that a low dose of METH promoted spine formation, synaptic number increase, post-synaptic density length elongation, and memory function. A high dose of METH induced synaptic degeneration, neuronal number loss, and memory impairment. This change is produced by affecting the activity of ras-related C3 botulinum toxin substrate 1 (Rac1) and cell division control protein 42 homolog.

Developing a predictive model that can classify and characterize the brain-based biomarkers for predicting METH addicts may directly lead to improved treatment outcomes. Zhou et al. applied the support vector machine (SVM)-based classification method to resting-state functional magnetic resonance imaging (rs-fMRI) data obtained from individuals with methamphetamine use disorder (MUD) and healthy controls (HCs) to identify brain-based features predictive of MUD. The results of this study not only indicate that MUD-related FC alterations were predictive of group membership but also suggest that machine-learning techniques could be used for the identification of MUD-related imaging biomarkers.

Overall, the six articles provide valuable insights into the neural pathophysiological mechanisms of neuropsychiatric disorders underlying substance abuse. The articles highlight the importance of understanding the complex interplay between neurotransmitters, neuroplasticity, spine plasticity, and behavioral sensitization in substance abuse. These insights can inform the development of more effective prevention and treatment strategies for individuals with substance use disorders.

## Author contributions

All authors listed have made a substantial, direct, and intellectual contribution to the work and approved it for publication.
